# Functional Imaging of the Human Brainstem during Somatosensory Input and Autonomic Output

**DOI:** 10.3389/fnhum.2013.00569

**Published:** 2013-09-17

**Authors:** Luke A. Henderson, Vaughan G. Macefield

**Affiliations:** ^1^Department of Anatomy and Histology, University of Sydney, Sydney, NSW, Australia; ^2^School of Medicine, University of Western Sydney, Sydney, NSW, Australia

**Keywords:** trigeminal nuclei, dorsal column nuclei, pain, sympathetic nerve activity, rostral ventrolateral medulla

## Abstract

Over the past half a century, many investigations in experimental animal have explored the functional roles of specific regions in the brainstem. Despite the accumulation of a considerable body of knowledge in, primarily, anesthetized preparations, relatively few studies have explored brainstem function in awake humans. It is important that human brainstem function is explored given that many neurological conditions, from obstructive sleep apnea, chronic pain, and hypertension, likely involve significant changes in the processing of information within the brainstem. Recent advances in the collection and processing of magnetic resonance images have resulted in the possibility of exploring brainstem activity changes in awake healthy individuals and in those with various clinical conditions. We and others have begun to explore changes in brainstem activity in humans during a number of challenges, including cutaneous and muscle pain, as well as during maneuvers that evoke increases in sympathetic nerve activity. More recently we have successfully recorded sympathetic nerve activity concurrently with functional magnetic resonance imaging of the brainstem, which will allow us, for the first time to explore brainstem sites directly responsible for conditions such as hypertension. Since many pathophysiological conditions no doubt involve changes in brainstem function and structure, defining these changes will likely result in a greater ability to develop more effective treatment regimens.

## Introduction

Since the advent of human functional Magnetic Resonance Imaging (fMRI) in the early 1990s, thousands of scientific investigations have explored brain activation patterns during various challenges. However, only a few investigators have explored brainstem function in humans using fMRI techniques and a few have even extended this into exploring regional activation patterns within the spinal cord. There a numerous factors that hinder human brainstem exploration using fMRI, including the intricate parcellation of regional functionality so that small voxel sizes need to be employed, the resulting low signal to noise ratios of small voxels, susceptibility due to structures such as the pontine cistern, the pulsatile signal from cerebrospinal fluid, and respiratory related brainstem movement.

**Figure 1:**
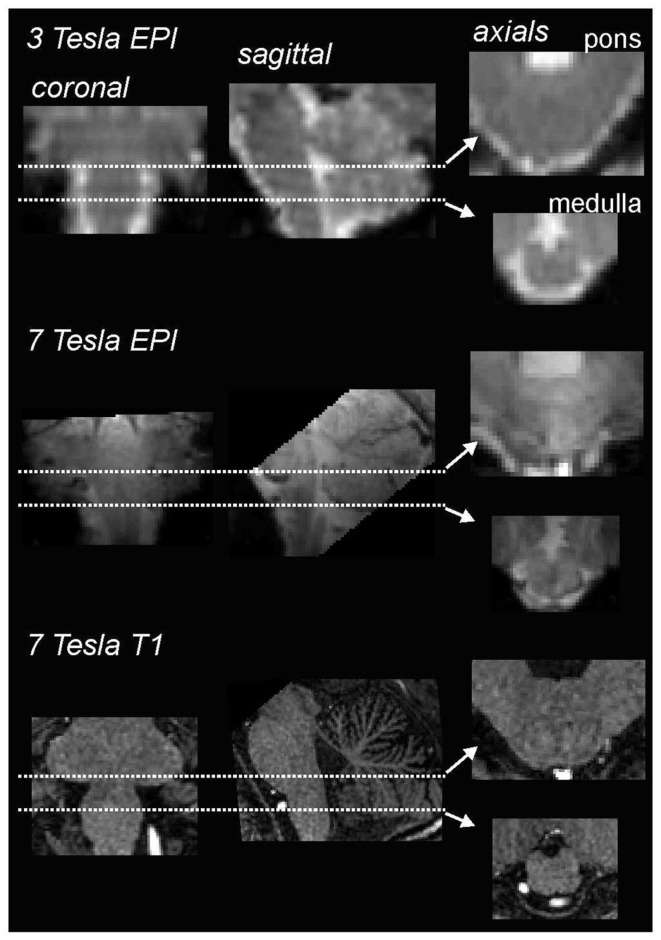
**Comparison of coronal, sagittal, and axial echo-planar images (EPI) through the medulla collected using a 3 T (top row) and 7 T (middle row) MRI scanner collected in the same individual**. Note that the images collected using the 7 T scanner have finer spatial resolution and greater gray/white differentiation. The bottom row shows a series of T1-weighted anatomical images also collected using a 7 T MRI scanner.

However, steady improvements in signal to noise ratios, due to improved scanner hardware, software, and increases in magnetic field strengths, the development of scanning techniques that lower signal dropout in susceptible regions, such as the basis pontis, as well as improved image analysis techniques – particularly software that allows improved spatial normalization of brainstem structures – have resulted in greatly improved accuracy and feasibility of brainstem fMRI imaging. Using a 3 T MRI scanner, we have found that, over the past 7 years, improvements in signal:noise have resulted in the ability to acquire images with significantly greater spatial resolution, i.e., raw voxel sizes have decreased from approximately 3 to 1.5 mm^3^. Of course the development of human MRI scanners with even higher field strengths, e.g., 7 T, has also resulted in improved spatial resolution, making it possible to collect images with raw voxel sizes of 1 mm^3^ (Figure 1). In addition to greater spatial resolution, the ability to compare signal intensity changes between subjects relies heavily on the accuracy of spatial normalization software. The development of brainstem-specific normalization software has allowed for greater accuracy between subjects and greatly improved the feasibility and accuracy of brainstem fMRI, particularly in studies involving comparisons within and between groups of subjects. In a recent article, Beissner et al. (2011) compared various brainstem imaging analysis techniques and found that five methods were found to be sensitive for brainstem activation, including analysis using various spatial smoothing kernels and spatial normalization in various software packages.

Given these improvements in brainstem fMRI image acquisition and processing, we are now in a position where brainstem function in humans can be explored with greater accuracy and ease. This is extremely important, since it is likely that functional changes in specific brainstem regions underlie the expression of many significant clinical conditions. For example, investigations in experimental animals have revealed that changes within the dorsal horn of the spinal cord or trigeminal nuclear complex in the brainstem are important for the expression of chronic pain (D’Mello and Dickenson, 2008). Additionally, many animal studies have defined the brainstem circuitry responsible for the generation of spontaneous sympathetic drive, such as the nucleus of the solitary tract, caudal, and rostral ventrolateral medulla (Dampney, 1994; Colombari et al., 2001). It is possible that activity changes within these brainstem regions are at least partially responsible for generating the increase in sympathetic drive associated with conditions like essential hypertension and obstructive sleep apnea. Although animal work has greatly enhanced our understanding of brainstem function, for the main part they have been performed in anesthetized, and often artificially ventilated preparations, and in disease models that do not precisely mimic those seen in humans. Given this, it is important that we begin to explore brainstem function in awake humans, to understand both normal physiology and the disturbed control seen in various pathophysiological states.

Over the past decade, we and others have been exploring human brainstem function and structure in healthy individuals and in various patient populations. The following sections will describe some of the recent results we have obtained in healthy individuals in which we have explored brainstem processing of somatosensory stimuli and sympathetic motor output. These provide the basis for exploring changes in brainstem function associated with conditions such as chronic pain and hypertension.

## Somatosensory Processing

### Noxious and non-noxious somatosensory processing in the orofacial system

Brainstem imaging provides us with the unique opportunity to investigate the processing of both noxious and non-noxious somatosensory information from the orofacial region. It is well known that primary afferent neurons carrying thermoreceptive and nociceptive somatosensory information from the orofacial region synapse in the spinal trigeminal nucleus (SpV), which extends throughout the lateral medulla and into the upper cervical spinal cord (Sessle, 2000). In contrast, orofacial discriminatory touch information is processed in the principle or chief sensory trigeminal nucleus, which lies within the dorsolateral pons. This differential afferent termination pattern allows for the investigation of nociceptive versus non-nociceptive processing within the human brainstem using high resolution fMRI.

Chronic pain, i.e., pain that extends beyond the expected period of healing and for more than 3 months, has a significant detrimental impact on an individual’s life. Since most forms of chronic pain are resistant to currently available treatments, they often persist for years and even decades. The lack of effective treatment regimens results from the fact that little is known about the underlying mechanisms, particularly those that result from nervous system damage (neuropathic pain). Given that animal evidence suggests that changes at the level of the primary synapse are involved in the generation and/or maintenance of some forms of chronic pain, and that in humans some pain treatments are thought to exert their analgesic effects via actions on the primary synapse (e.g., opiates, transcutaneous electrical nerve stimulation), it is important that we begin to explore the processing of noxious information at the primary synapse so that the underlying mechanisms of chronic pain are understood and more effective treatment regimes are developed.

**Figure 2:**
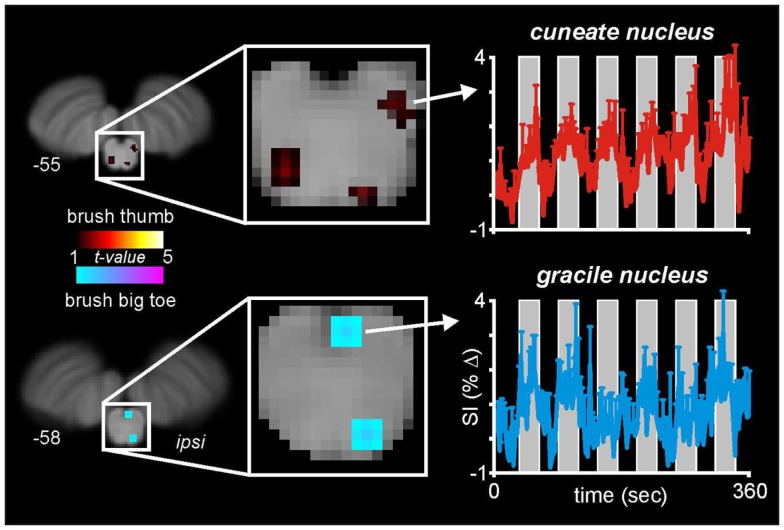
**Medullary activation during repeated innocuous brushing of the thumb and big toe in five subjects**. Note that thumb brushing activates a region of the ipsilateral dorsal medulla that is lateral and marginally rostral to that region activated by brushing of the big toe. These activation patterns are consistent with the well-described termination patterns of non-noxious somatosensory afferent neurons, i.e., the cuneate and gracile nuclei. Note that during each of the six brushing period (vertical gray bars), the mean (±SEM) percentage change in signal intensity increases. The slice locations in Montreal Neurological Institute space are shown at the lower left of each section.

With this in mind, we and others have begun to use fMRI to explore neural activation patterns during acute noxious and non-noxious somatosensory stimulation in healthy individuals. In a recent series of investigations, we measured brain activity using a 3 T MRI scanner during non-noxious somatosensory activation evoked by lightly brushing various parts of the body in healthy individuals (Wrigley et al., 2009; Henderson et al., 2011; Gustin et al., 2012). Using Blood Oxygen Level Dependant (BOLD) imaging, we collected 130 fMRI volumes covering the entire brain, during which we brushed the big toe or thumb using a repeated “On-Off” paradigm (seven Off, six On periods). Although the aim of this investigation was to explore changes within the primary somatosensory cortex, in five individuals our fMRI scans also encompassed the caudal medulla, including the level at which non-noxious somatosensory afferents from the body terminate. In a preliminary investigation we explored medullary signal changes during thumb and toe brushing to determine if we could identify activation of the cuneate and gracile nuclei, respectively. Using brainstem-specific software (SUIT toolbox in SPM8) (Diedrichsen et al., 2011) we isolated and normalized the brainstem, applied global signal detrending (Macey et al., 2004) and then determined significant patterns of signal intensity increases during each brushing period using a repeated box-car model. A second-level conjunction analysis (*p* < 0.005, uncorrected for multiple comparisons) revealed the well-described somatotopic organization of non-noxious somatosensory termination patterns within the brainstem. That is, innocuous brushing of the big toe resulted in activation of the region of the ipsilateral gracile nucleus, whereas brushing of the thumb activated the region of the ipsilateral cuneate nucleus (Figure 2). It can be seen in Figure 2 that signal intensity increased during each brushing period and subsequently returned toward baseline levels during the rest periods. A similar activation pattern during innocuous stimulation of the upper limb has been shown previously (Ghazni et al., 2010). We also found signal intensity increases during both brushing paradigms in the region of the lateral reticular nucleus. It is known from experimental animal investigations that lateral reticular nucleus neurons receive ascending inputs from the dorsal horn and respond to noxious and non-noxious somatosensory stimulation (Kitai et al., 1967). Although the precise role of this region remains unknown, it projects to the cerebellar vermis and may play a role in co-ordinating movement, although a role for this region in cardiovascular regulation has also been described (Thomas et al., 1977). In addition to a somatotopic representation within the lower brainstem, we have previously shown a similar organization within the relevant recipient region of the thalamus and primary somatosensory cortex (Wrigley et al., 2009; Gustin et al., 2012).

**Figure 3:**
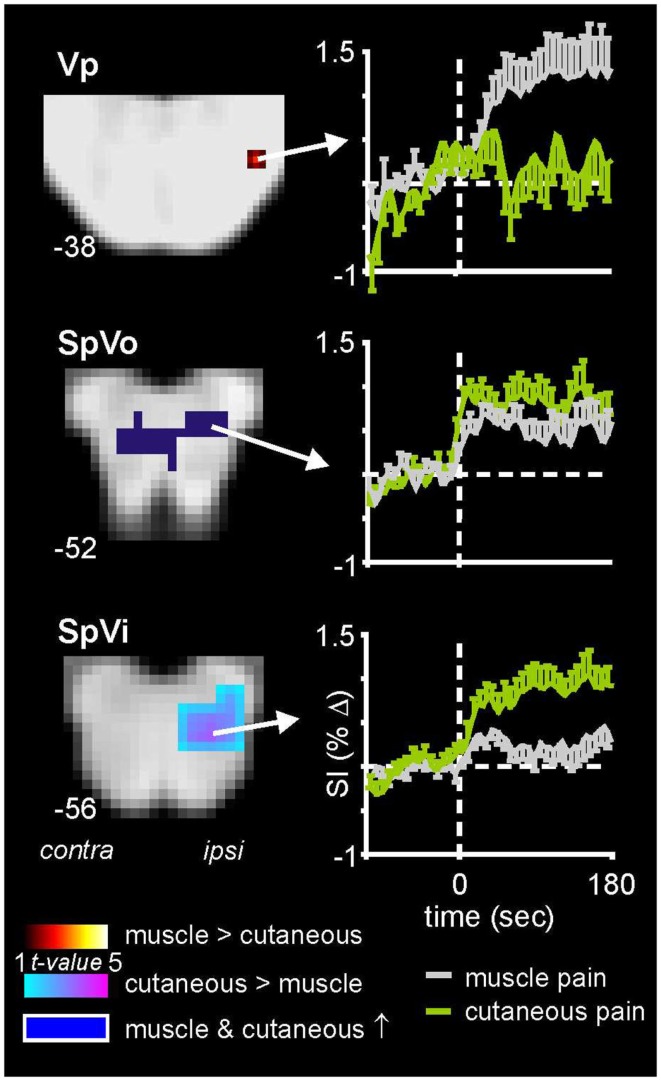
**Brainstem activation during noxious stimulation of the masseter muscle and the skin overlying the masseter muscle in 18 healthy subjects**. Note that whereas cutaneous and muscle pain evoked activation of both the caudalis (data not shown) and oralis divisions of the spinal trigeminal nucleus (SpVo), only cutaneous pain activated the interpolaris division of SpV (SpVi). Furthermore, only muscle pain activated the principle trigeminal nucleus (Vp). The slice locations in Montreal Neurological Institute space are shown at the lower left of each section. Plots of mean (±SEM) percentage change in signal intensity (SI) over time are shown for each region. The vertical dashed lines indicate the onset of either the cutaneous (green) or intramuscular (gray) injections of hypertonic saline.

As well non-noxious somatosensory activation patterns, we have explored brainstem activity evoked by noxious somatosensory stimulation involving two separate methods: acute muscle pain – evoked by a 0.5 ml injection of hypertonic (5%) saline into the belly of the right masseter muscle – and acute cutaneous pain, evoked by injection of hypertonic saline into the skin overlying the masseter muscle (Nash et al., 2009). We used the same image collection parameters and a similar analysis procedure as that employed for the experiments involving non-noxious somatosensory stimulation. We know from animal work that the SpV is divided into three major divisions: oralis, interpolaris, and caudalis, from rostral to caudal (Sessle, 2000). Although the roles of these subnuclei have begun to be explored in experimental animals, it remains unknown if differences in noxious processing occur in these different subnuclei in humans. In our investigations, we found that although acute pain evoked significant increases in signal intensity within the ipsilateral SpV, the pattern of activation differed depending on whether the noxious stimulus was delivered to skin or muscle. Whereas both cutaneous and muscle pain activated the caudalis and oralis divisions of SpV, only cutaneous pain activated the interpolaris division (Figure 3) (Nash et al., 2009). Furthermore, we found that orofacial muscle pain – but not cutaneous pain – evoked increases in signal intensity in the region of the pons that was also activated by innocuous lip brushing, i.e., the principal sensory nucleus (Vp). It is possible that these differential patterns of activation reflect differences in higher processing of the perceptual, emotional, and motoric consequences of deep compared with superficial pain (Lewis, 1942).

In addition to differential activation patterns according to the type of tissue stimulated, it is well known that the caudalis division of SpV is somatotopically organized, with inputs from the three trigeminal nerve divisions – ophthalmic, maxillary, and mandibular – being arranged in a rostrocaudal fashion. Of course, this termination pattern is responsible for the unique “onion skin” pattern of pain and temperature perception loss that occurs in humans following a lesion to a discrete rostrocaudal level of the spinal trigeminal caudalis nucleus. Remarkably, somatotopic organization of the SpV, as well as the trigeminal ganglion, has been reported using fMRI. Over 10 years ago, Borsook et al. (2003) assessed changes in fMRI signal intensity within the trigeminal ganglion during innocuous brushing and noxious thermal stimulation of the face within the three trigeminal nerve receptive fields. Remarkably, they found that stimulation in these different receptive fields evoked differential activation patterns within the trigeminal ganglion, consistent with the known afferent locations derived from anatomical investigations.

Even more remarkable are the recent investigations that use fMRI to explore activity changes within the dorsal horn of the spinal cord. For example, using a 3 T scanner, Nash et al. (2013) collected BOLD images of the cervical spinal cord using a spiral in-out image sequence with retrospective correction for physiological noise, to investigate activation patterns within the cervical spinal cord during noxious stimulation of the skin overlying the deltoid muscle and thenar eminence. They found that noxious stimulation evoked increases in signal intensity in the region of the ipsilateral dorsal horn at the relevant spinal cord level (deltoid: C4–C5; thenar eminence: C6–C7). These human fMRI studies show that it is now possible to investigate activation patterns throughout the entire “pain” pathway, from the sensory ganglion and dorsal horn/SpV, rostrally to the thalamus and up to cortical regions. This opens up the possibility to explore changes at all levels of the nervous system in individuals with, for example, chronic pain. Such an improvement in our understanding of the underlying neurophysiological processes responsible for the generation of acute pain may ultimately lead to better treatment of chronic pain.

## Autonomic Output

The brainstem contains several nuclei involved in autonomic control, the most studied being those which regulate the cardiovascular system. Since hypertension is a significant risk factor for the development of a number of serious medical conditions, and neurogenic hypertension is brought about by an increase in sympathetically mediated constriction of muscle (and splanchnic) vascular beds, it is critical that we begin to explore the underlying circuitry responsible for generating sympathetic vasoconstrictor drive in humans. Although thousands of investigations have defined brainstem and higher circuitry involved in generating and/or modulating arterial pressure, heart rate, and sympathetic nerve activity in anesthetized experimental animals, relatively few have explored this circuitry in awake human subjects (Macefield et al., 2006; Macefield and Henderson, 2010; Sander et al., 2010). Additionally, despite much experimental focus on the causes of hypertension in humans, the underlying mechanisms responsible for most hypertensive conditions remains almost completely unknown. Indeed, essential hypertension, which defines about 95% of all hypertensive patients, has no identifiable cause, although a number of risk factors have been identified. Furthermore, despite the development of antihypertensive drugs, in most individuals, hypertension and its associated risk factors remain uncontrolled (Messerli et al., 2007). If effective control of hypertension is to be obtained in all individuals with essential hypertension or in individuals with other conditions associated with high blood pressure, such as obstructive sleep apnea (Carlson et al., 1993), an understanding of the underlying circuitry responsible for generating baseline sympathetic drive needs to be obtained in normotensive individuals before we begin to explore potential changes in this circuitry in individuals with various forms of hypertension.

Muscle sympathetic nerve activity (MSNA) can be recorded directly in via tungsten microelectrodes inserted percutaneously into a peripheral nerve in awake human subjects (microneurography). Developed over 40 years ago the technique has provided a wealth of information on the human sympathetic nervous system and its role in the control of blood pressure (Vallbo et al., 2004). It is well established that there is a tight relationship between dynamic changes in arterial pressure and MSNA through the operation of the baroreflex. Spontaneous variations in arterial pressure are detected by high-pressure arterial baroreceptors in the aortic arch and carotid sinuses, which send signals to the medulla to evoke opposing changes in MSNA. This reflex acts to cushion the original change in pressure and maintain resting arterial pressure at a stable level. The neural circuitry responsible for the baroreflex has been extensively investigated over many decades in anesthetized experimental animals. Baroreceptor afferents, traveling in the glossopharyngeal and vagus nerves, project to the caudal region of the nucleus tractus solitarius (NTS) which in turn provides excitatory drive onto tonically active inhibitory neurons in the region of the caudal ventrolateral medulla (CVLM). This region then inhibits excitatory neurons within the region of the rostroventrolateral medulla (RVLM), which contains premotor neurons which descend down the spinal cord to synapse with sympathetic preganglionic neurons within the intermediolateral cell column throughout the thoracic and upper lumbar cord (Dampney, 1981). These investigations have also revealed that activity of the RVLM is critical for the maintenance of resting vasomotor tone and arterial pressure, since RVLM destruction results in precipitous falls in resting arterial pressure (Dampney et al., 1982). Furthermore, it appears that the RVLM provides the major output nucleus from which almost all brain regions, including the cerebral cortex, control arterial pressure. For example, during physical and psychological stressors, higher brain regions such as the cingulate, insular, and prefrontal cortices project to the RVLM to alter arterial pressure and sympathetic drive (Dampney et al., 2002; Gabbott et al., 2005). Given this, the RVLM and its contacts are likely candidates in which altered structure and function may result in hypertension in various medical conditions.

Whilst the medullary circuitry responsible for the baroreflex has been extensively studied in experimental animals, for the vast majority of these studies the animal was anesthetized and often ventilated. Although this reflex may work the same way in awake humans, it is known that in at least some brain regions, anesthesia has a significant effect on the cardiovascular response to stimulation. For example, chemical stimulation of the nucleus raphe obscurus in the medulla evokes increases in arterial pressure and heart rate under halothane anesthesia, yet decreases under urethane anesthesia (Henderson et al., 1998; Heslop et al., 2002). Furthermore, given the lack of effective hypertensive animal models, at least ones that mimic hypertension in conditions such as obstructive sleep apnea and essential hypertension, it is important that we investigate this circuitry in normotensive awake humans such that we can eventually elucidate changes in brain structure and function in individuals with various forms of hypertension.

### Brainstem activity during evoked changes in muscle sympathetic nerve activity

Over the past decade a few investigators have begun to explore brain sites responsible for changes in sympathetic drive. We and others have indirectly measured human baroreflex functioning by using maneuvers such as lower-body negative pressure (Kimmerly et al., 2005), Valsalva maneuvers (Harper et al., 1998; Henderson et al., 2002), static hand grip (Sander et al., 2010), and maximal inspiratory apneas (Macefield et al., 2006). Although these studies were not designed or analyzed with brainstem-specific techniques, some did find that increased sympathetic drive was associated with increased signal intensity in the region of the RVLM, as well as a number of higher brain regions. However, there are some considerable methodological issues associated with these maneuvers that increase sympathetic nerve activity. While the sympathetic responses to challenges such as maximal inspiratory apneas and Valsalva maneuvers are relatively large and robust, they are also associated with significant subject movement, and may require significant skeletal motor activity to perform the actual task and often large and rapid increases in arterial pressure. Even though these issues can be somewhat overcome by various analytical procedures, they do complicate the interpretation of the data. Furthermore, in these investigations, MSNA (and often arterial pressure) changes were recorded during a separate session, a situation that does not allow for subtle difference in brain function to be assessed.

**Figure 4:**
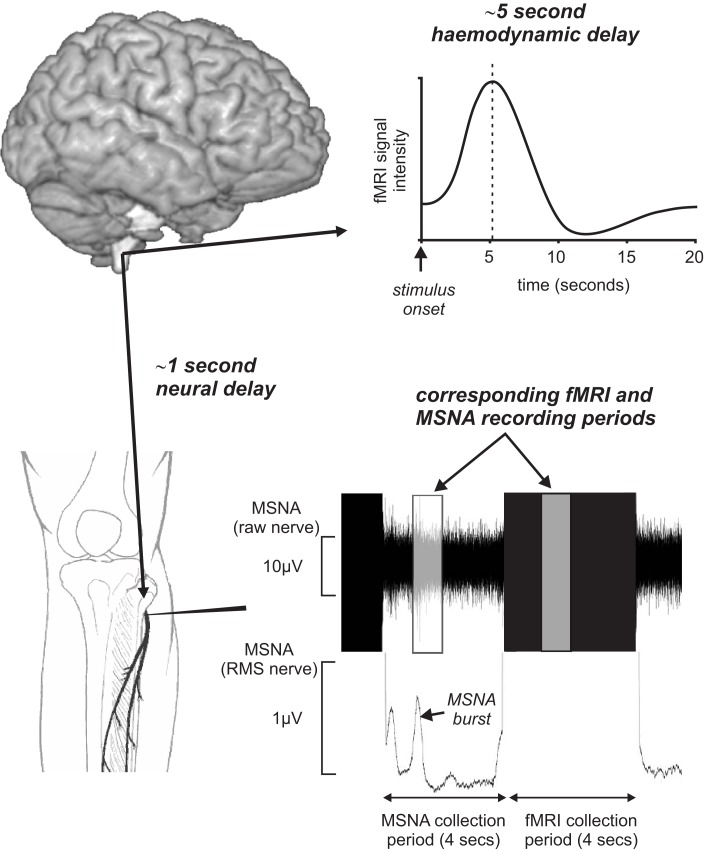
**Concurrent recording of muscle sympathetic nerve activity (MSNA) and brain fMRI using a 4 s MSNA collection period followed by a 4 s brain fMRI collection period**. Given an fMRI hemodynamic delay of approximately 5 s and the delay of approximately 1 s for MSNA traffic to travel from the brain to the recording electrode, brain activity during the MSNA collection period was reflected in signal intensity changes during the following 4 s period. RMS, root mean square.

In an attempt to remove these potential confounding issues, we have recently managed to successfully record brainstem fMRI and MSNA (using microneurography) concurrently in awake normotensive human at rest (Macefield and Henderson, 2010). By using a scan repetition time of 8 s and implemented a 4 s image collection period followed by a 4 s rest period we were able to collect brainstem BOLD images and MSNA in concurrent 4 s periods. This protocol was chosen to take advantage of the temporal delays inherent in BOLD imaging. Since the peak of the hemodynamic delay is estimated to lag neural activity by approximately 5 s (Logothetis et al., 2001), and given that we need to allow approximately 1 s for a muscle vasoconstrictor volley to travel from the brainstem to the peripheral recording site at the knee (Fagius and Wallin, 1980), the changes in BOLD signal intensity within the brainstem would reflect changes in neural activity associated with emission of sympathetic volleys recorded in the previous 4 s epoch (Figure 4). Furthermore, we collected axial slices sequentially from caudal to rostral so that the timing of each slice could be related to the MSNA recording.

### Brainstem activity during spontaneous fluctuations in muscle sympathetic nerve activity

**Figure 5:**
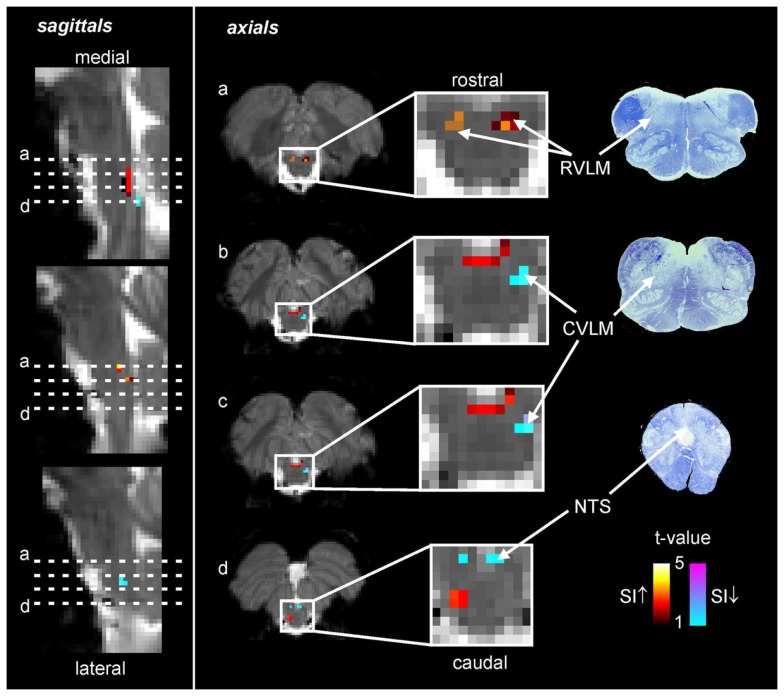
**Functional magnetic resonance imaging (fMRI) signal intensity changes correlated to spontaneous fluctuations in muscle sympathetic nerve activity (MSNA) in eight subjects**. Increases in signal intensity with increases in MSNA are coded by the hot color scale and signal decreases with the cool color scale and are overlaid onto a series of sagittal and axial fMRI slices from an individual subject. Myelin stained sections through the medulla are shown to the right. CVLM, caudal ventrolateral medulla; NTS, nucleus tractus solitaries; RVLM, rostral ventrolateral medulla; SI, signal intensity.

**Figure 6:**
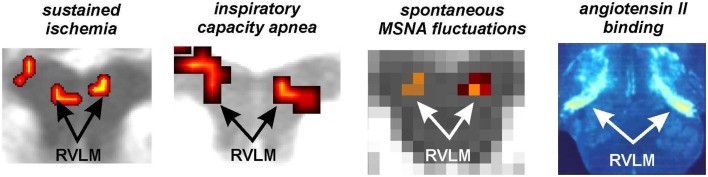
**Functional and anatomical localization of the human rostroventrolateral medulla (RVLM)**. From left to right: bilateral increase in RVLM functional magnetic resonance imaging (fMRI) signal intensity during a sustained muscle ischemia (Sander et al., 2010), maximal inspiratory breath-hold (Macefield et al., 2006), spontaneous fluctuations in muscle sympathetic nerve activity (MSNA) (Macefield and Henderson, 2010), anatomical identification of the RVLM using the binding of Angiotensin II receptors (reproduced with permission from Allen et al., 1998).

Using the protocol described above, we explored the medullary circuits responsible for the baroreflex by measuring regional brainstem activity changes during small, spontaneous changes in MSNA during 10 recording sessions in 8 awake normotensive humans. Using brainstem-specific analysis techniques, we determined which brainstem regions displayed high or low signal intensity during periods of high MSNA. We found that small increases in MSNA were associated with fMRI signal intensity increases in the RVLM and signal decreases within the regions of the CVLM and NTS (Figure 5). That is, significant correlations between regional brainstem signal intensity and MSNA were found to occur in those regions previously described as being responsible for baroreflex functioning in anesthetized experimental animals, the RVLM, CVLM, and NTS (Macefield and Henderson, 2010). The increase in signal intensity within the region of the RVLM coincides with similar activation patterns evoked by maximal inspiratory capacity apneas and sustained hand grip, challenges which are also associated with significant increases in MSNA (refs). Furthermore, these activations lie in the same region defined anatomically as the RVLM by the binding of angiotensin receptors (Allen et al., 1998) (Figure 6).

The identification of sites within the brainstem, and in particular the medulla, are the first step in elucidating brainstem and higher changes associated with hypertensive conditions. It has been shown that essential hypertension and obesity (a risk factor for hypertension and obstructive sleep apnea, a condition with comorbid hypertension) are associated with altered brain structure including gray matter volume changes in areas such as the insula cortex and hippocampus (Macey et al., 2002; Taki et al., 2008). It is possible that in these and other conditions, changes in higher brain structures that occur either before or during the early pre-clinical stages of the condition, result in altered influences over the RVLM, i.e., reduced inhibition or increased excitation, resulting in increased RVLM activation and a resulting increase in arterial pressure and on-going MSNA. Future investigations should firstly examine changes within the region of the RVLM and subsequently changes in higher brain structures that may influence RVLM function.

### Brainstem activity during evoked changes in skin sympathetic nerve activity

Finally, in addition to work exploring brainstem sites responsible for evoked and spontaneous changes in MSNA, recent investigations have also explored changes in brain activity, including the brainstem, during changes in skin sympathetic nerve activity (SSNA) or sweating, a rather sluggish marker of SSNA. The sympathetic innervation of the skin in humans primarily subserves thermoregulation, by constricting or dilating cutaneous blood vessels and increasing or decreasing the release of sweat. In thermoneutral conditions, resting SSNA is primarily related to the level of arousal and emotional state (Hagbarth et al., 1972). We recently recorded whole brain fMRI and SSNA concurrently in 13 healthy individuals whilst viewing emotionally charged images (erotica and mutilation) (Henderson et al., 2012). We found that viewing emotionally charged images increased SSNA, and that this SSNA increase was associated with altered activity in brain regions such as the amygdala, pons, thalamus, nucleus accumbens, cerebellar cortex, and precuneus. Additionally, we recently made concurrent SSNA and fMRI recordings in 13 awake human subjects to identify brain regions associated with the generation of spontaneous SSNA (James et al., 2013). Spontaneous SSNA increases were associated with signal changes in the thalamus, insula, cingulate cortex, and precuneus.

In recent publications by McAllen et al., brainstem activation patterns were explored during changes in skin temperature and during psychological stressors. They found that skin cooling evoked fMRI signal intensity increases in rostral medullary raphe region, a region shown to contain thermoregulatory neurons in rodents (McAllen et al., 2006). Further, they found that thermal (whole-body heating, 11 subjects) and psychological stressors (Stroop test; 11 subjects), evoked increased sweating and were associated with increased signal intensity in the rostral lateral midbrain and in the rostral lateral medulla (Farrell et al., 2013). Although the site of activation within the lateral medulla was located close to that which we previously identified as the RVLM, the authors propose that the activation is located in the region between the facial nuclei and pyramidal tracts, an area that corresponds to a neuronal group found to evoke sweating in experimental animals.

## Conclusion

While the brainstem – and especially the medullary component – present significant difficulties for neuroimaging, there is a clear need to understand the functional organization of this phylogenetically ancient structure. With the advent of higher field strengths (e.g., 7 T), and improvements in spatial resolution, our capacity to understand the operation of the brainstem will increase enormously. Nevertheless, it is remarkable that individual nuclei can be functionally identified even at 3 T, and that the microcirculation within the brainstem allows BOLD imaging in such a small volume.

## Conflict of Interest Statement

The authors declare that the research was conducted in the absence of any commercial or financial relationships that could be construed as a potential conflict of interest.
